# Printed Lithography of Graphene-Perovskite Quantum Dot Hybrid Photodetectors on Paper Substrates

**DOI:** 10.1021/acsami.4c18102

**Published:** 2025-01-20

**Authors:** Yujia Li, Yining Zhao, Alfonso Ruocco, Mingqing Wang, Bing Li, Shahab Akhavan

**Affiliations:** † Institute for Materials Discovery, University College London, London WC1E 7JE, U.K.; ‡ Department of Chemistry, University College London, London WC1E 7JE, U.K.; ¶ Optical Networks Group, University College London, London WC1E 6BT, U.K.

**Keywords:** Paper, Printing, Photodetectors, Graphene, Perovskite, Quantum Dots

## Abstract

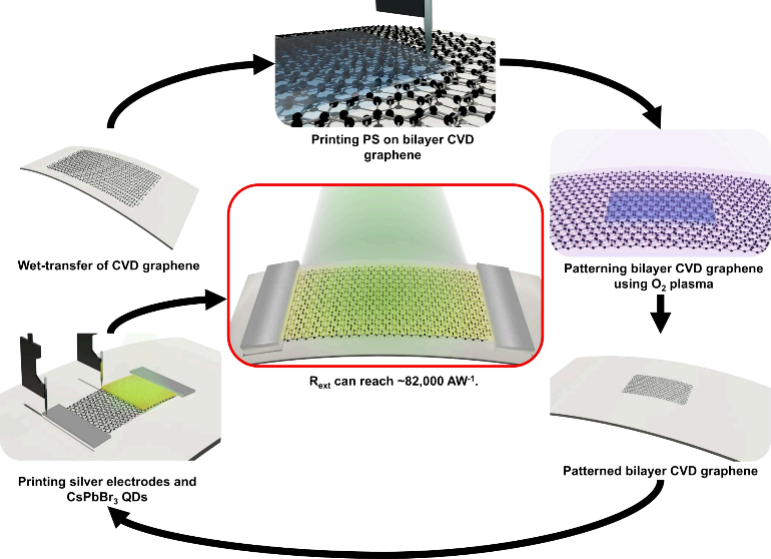

Paper is an ideal platform for creating flexible and eco-friendly electronic systems. Leveraging the synergistic integration of zero- and two-dimensional materials, it unfolds a broad spectrum of applications within the realm of the Internet of Things (IoT), spanning from wearable electronics to smart packaging solutions. However, for paper without a polymer coating, the rough and porous nature presents significant challenges as a substrate for electronics, and the absence of well-established fabrication methods further hinders its application in wearable electronics. In this study, we present photodetectors (PDs) on a paper substrate composed of graphene and CsPbBr_3_ perovskite quantum dots (PQDs). Hybrid structures that combine PQDs with graphene offer a promising approach for PDs. These structures benefit from robust quantum confinement in PQDs alongside improved light interaction, tunable spectra, high absorption coefficients, and an enhanced photoconductive gain mechanism in graphene, all at ambient conditions. We use a microplotter for the lithographic printing of graphene, silver electrodes, and PQDs, to fabricate PDs on paper. These PDs have an external responsivity of ∼82,000 AW^–1^ at 520 nm for an operating voltage ⩽1 V. The external responsivity is 3 orders of magnitude higher than state-of-the-art paper-based PDs. Under bending at L_0_/L = 1.15 (L_0_ is the arc length and *L* is the chord length) and after 600 bending cycles, the external responsivity is maintained up to 80%. Thus, the combination of zero- and two-dimensional materials via microplotting on a paper substrate shows promise for wearable and flexible applications.

## Introduction

Derived from renewable and abundant resources, paper-based electronics are anticipated to alleviate landfill issues and reduce environmental impacts associated with recycling processes. Various PDs have been developed with paper-based PDs offering cost efficiency and enhanced flexibility over other substrates. However, they present significant challenges, such as low external responsivity (R_*ext*_) and high operating voltages.^1−3^ Paper substrates are seldom used in their native state, often requiring additional coatings or planarization to smooth paper substrates due to their inherent porosity and surface roughness.^4^ This porosity leads to elevated roughness, limited stability, reduced resistance to thermal and humidity fluctuations, and high hygroscopicity, all of which can adversely affect the electrical properties of overlying devices.^5−8^ These factors contribute to the absence of established, reliable fabrication methods, impeding widespread industrial adoption. Additionally, the R_*ext*_ of paper-based PDs is typically much lower than that of devices on rigid substrates for example, 10^9^ AW^–1^ at 0.5 V under 598 nm illumination on Si/SiO_2_.^9^ Conventional fabrication techniques optimized for rigid substrates such as Si/SiO_2_ and GaAs are incompatible with paper, limiting its use in wearable applications.

Various printing techniques, including dip coating, inkjet printing, and microprinting, have been employed in paper-based electronics, each with distinct advantages and limitations. Dip coating is a simple, scalable method suitable for creating uniform films over large areas, as demonstrated by Maity et al. in fabricating MAPbBr_3_-based flexible detectors.^10^ However, it lacks precision for detailed patterns and may result in nonuniform coating thickness,^11^ and evaporation of metals to make electrodes.^10^ Inkjet printing offers a balance of precise patterning, minimal material waste, and scalability but necessitates careful optimization of ink and surface properties for consistent performance;^12^ for instance, Kara et al. improved resolution to 50 μm by adding α-terpineol to PbS QD ink.^13^ However, its performance is limited due to the coffee ring effect, blocking nozzles, and stringent requirements for the Weber, Reynolds, and Ohnesorge numbers of the ink to form well-controlled droplets. Furthermore, the implementation of complex e-beam patterning of CVD SLG and the evaporation of Au electrodes added significant complexity and expense to the process.^13^ Considering the aforementioned limitations, the microplotting process emerges as a viable alternative. Microprinting has the ability to print a variety of viscous inks, which gives great potential for flexible, wearable applications. enabling intricate patterns with features as small as 20 μm using picoliter droplets dispensed via controlled ultrasonics.^14,15^

Two-dimensional (2d) materials have gained significant attention in PDs due to their unique electronic, optical, and mechanical properties. Graphene and graphene-related materials (GRMs) are particularly suitable for various optoelectronic applications. Among synthesis methods like micromechanical exfoliation, liquid-phase exfoliation, and reduced graphene oxide (rGO), chemical vapor deposition (CVD) graphene stands out for scalable production of high-quality wearable PDs.^16,17^ CVD graphene exhibits fewer defects than dispersed graphene, reducing recombination centers that lower PD efficiency, and offers uniformity over large areas for consistent device performance.^18−20^ Its electron mobility exceeds 11 cm^2^·V^–1^·s^–1^ on flexible substrates, outperforming other scalable methods like electrochemical exfoliation.^21,22^ However, challenges include poor adhesion of printed 2D inks to flexible substrates without polymeric binders, which limit functionalities and degrade performance under bending.^23^ While CVD graphene roll-to-roll fabrication is becoming more economical, patterning still relies on complex techniques like optical and e-beam lithography, incompatible with paper substrates and cost constraints.^24^ Additionally, graphene lacks a mechanism to multiply charge carriers generated by single photons, leading to limited R_*ext*_ of approximately 0.01 AW^–1^ for single-layer graphene (SLG) PDs.^25^ PQDs are promising photoactive materials, due to their long charge-carrier diffusion lengths, strong absorption coefficients, and bandgap tunability via chemical composition control.^26−31^ As a result, integrating solution-processed PQDs with SLG, which exhibits a significantly higher mobility than PQDs, shows great potential for improving gain in light sensing and flexible applications.

In this project, we successfully developed paper-based PDs with exceptionally high R_*ext*_ values at low operating voltages by integrating CVD and microprinting techniques. Unlike existing paper-based electronics that require additional layers or surface treatments, we employed a scalable printed-lithography method directly on commercial glossy paper substrates.^32^ Using a microplotter, we performed lithographic patterning of transferred SLG on paper and directly printed PQDs and electrodes to fabricate the PDs. In contrast to common fabrication techniques on paper substrates that involve single or multiple steps of lithography or evaporation, our developed technology eliminates the need for such intricate and costly fabrication tools. By leveraging the strong absorption coefficients of PQDs and integrating them with SLG, we enhanced the photoconductive gain mechanism, achieving an R_*ext*_ of approximately 82,000 AW^–1^ at 520 nm with a bias voltage of 1 V. To our knowledge, this PQD/SLG PD demonstrates the highest R_*ext*_ among paper-based PDs to date, surpassing state-of-the-art devices by 3 orders of magnitude, as summarized in Table 1. The responsivity can be further increased by applying a higher source-drain voltage bias. This technology enables the efficient fabrication of channels and the creation of tailored electronic devices and circuits. It offers a promising route to overcome the limitations of traditional fabrication methods, paving the way for enhanced, large-area, sustainable electronic devices.

**Table 1:** Properties of Paper-Based Flexible PDs

Device type	Wavelength (nm)	Responsivity (AW^–1^)	Bias (V)	Bending cycles	Detectivity (Jones)	ref.
CsPbBr_3_ QDs/CVD graphene/paper	520	82,000	1	800 at L_0_/L = 1.15	7.3 × 10^10^	This work
ZnO/paper	254	12 × 10^–6^	10	400 at 30°	8.14 × 10^8^	(3)
a-Ga_2_O_3_/paper	254	3.1 × 10^–3^	-	100 at 12 cm^–1^	1.42 × 10^11^	(66)
WSe_2_ nanosheets-PANI/paper	670	1.726 × 10^–2^	-	400	7.48 × 10^9^	(62)
ZnS-MoS_2_/paper	554	17.85 × 10^–6^	-	-	-	(67)
ZnO/paper	354	0.06	9	150 at 60°	-	(68)
MAPbl_3_/paper	633	4.4 × 10^–3^	5	1000	-	(69)
WS_2_ nanosheets-graphene/paper	670	0.439	10	500	1.41 × 10^10^	(61)
PVK-MXene/paper	450	4.49 × 10^–2^	10	1500 at 60°	6.4 × 10^8^	(70)
WSe_2_ nanodots/paper	670	1.778 × 10^–2^	5	50	5.86 × 10^10^	(71)
CsPbCl_3_ nanocrystals/graphene/paper	405	520	1	500	-	(72)
MAPbBr_3_/paper	365	1.3	1	1000 at L_0_/L = 6	-	(51)
Au/CsPbBr_3_–PMMA/ITO/glass	400–510	3.7–5.2	5	-	-	(73)
CsPbBr_3_ nanoribbons-PCBM/quartz	504	18.4	10	-	-	(74)
CsPbBr_3_ nanonet films/glass	473	2.84	5	-	5.47 × 10^12^	(75)
CsPbBr_3_ nanowire/glass	473	0.3	0	-	1 × 10^13^	(76)
CsPbBr_3_ micro/nanoflake/SiO_2_	275	2.78	5	-	-	(77)

## Results and Discussion

**Figure 1:**
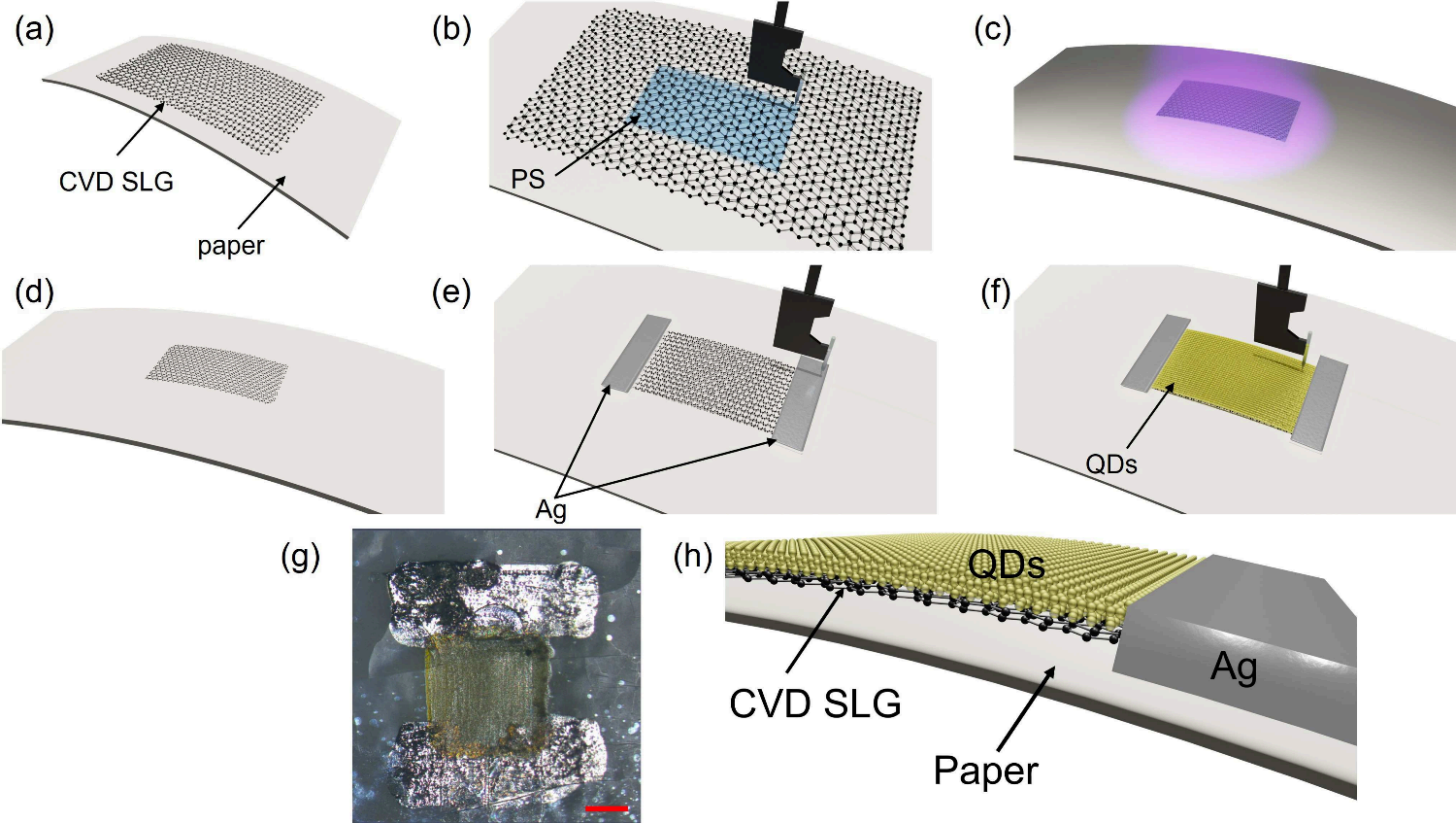
Fabrication process and schematic of the paper-based PQDs/SLG PD. (a) Transferred CVD SLG layers on paper. (b) Printed PS layer on CVD SLG layers. (c-d) Patterned CVD SLG layers via O_2_ plasma. (e) Printed Ag ink. (f) Printed PQDs layers. (g) Optical photograph of paper-based flexible PD. Scale bar: 500 μm. (h) Schematic of the flexible paper-based PD.

Here, we provide detailed fabrication steps for our flexible paper-based PDs composed of graphene and PQDs, as shown in Figure 1. Commercial glossy paper was used as the substrate, and CVD SLG was wet-transferred on the paper, as detailed in the Supporting Information (Figure S1). Unlike other studies that added layers or planarizations to smooth paper substrates, requiring complex high-temperature processes, we used commercial glossy paper. A flowchart of the wet-transfer procedure is illustrated in Figure S1. The CVD SLG on Cu was first spin-coated with polymethyl-methacrylate (PMMA) and then the PMMA/CVD SLG layer was separated from the Cu foil in ammonium persulfate (APS) solution. After the wet-transfer and overnight drying of SLG on a paper substrate, the PMMA was removed by acetone and isopropyl alcohol (IPA), as shown in Figure 1a. We repeated these steps to transfer the second layer of CVD SLG onto the paper substrate. The sheet resistances for different transferred CVD SLG layers on paper substrate were demonstrated in Table S1. The micropatterning of CVD graphene is achieved by printing a polystyrene (PS) protective layer on the surface of CVD graphene, as shown in Figure 1b. This process is followed by the O_2_ plasma etching (30 W and 20 s), as shown in Figure 1c. The PS layer is finally removed by soaking in acetone and IPA for 15 min, respectively, as shown in Figure 1d. Then the silver (Ag) ink is printed and annealed (150 °C for 2 h) at both ends of the channel of PD, as shown in Figure 1e, and then the PQDs are printed on the channel, as shown in Figure 1f. All these printing processes are completed using the Microplotter Proto, and the active area of the PQDs/SLG channel is approximately 7.31 × 10^–7^ m^2^ (length 884.6 μm, width 826.9 μm). We investigated the surface tension and contact angle of the PS ink, Ag ink, and PQDs ink on CVD graphene, as shown in Figure S2 and Table S2.

**Figure 2:**
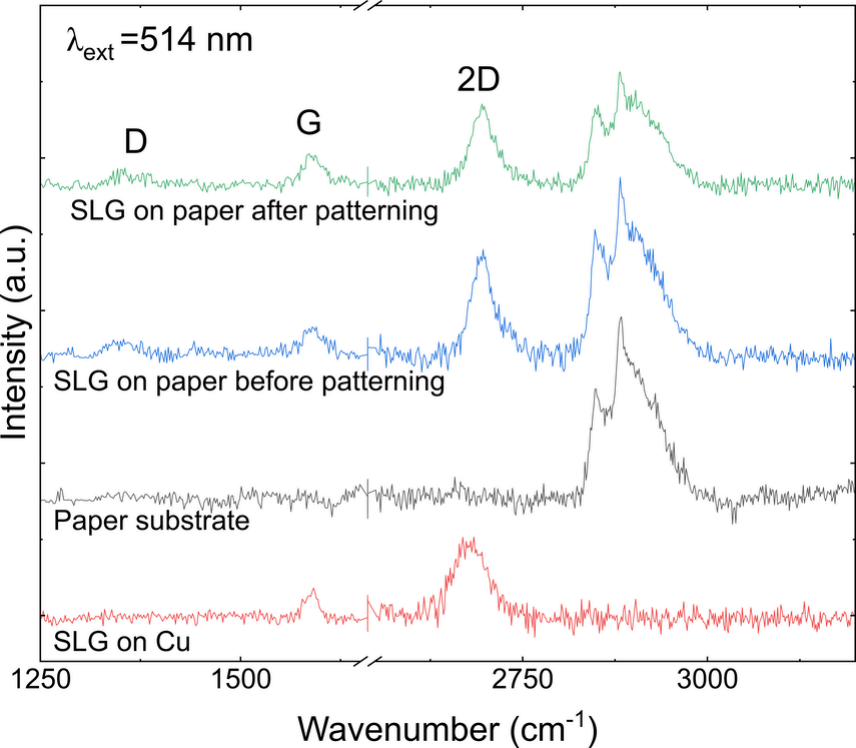
Raman spectra acquired from CVD graphene on Cu foil, paper substrate, and graphene on paper substrate before and after O_2_ plasma patterning. Spectra were normalized to have the same I(G).

The presence and quantity of CVD graphene were further studied by Raman spectroscopy. Raman spectra were acquired at 514 nm using a Renishaw InVia with a 100× objective and laser power <0.5 mW. Figure 2 shows the Raman spectrum (red) of the film as grown on Cu. The 2D peak is a single Lorentzian with fwhm(2D) ∼ 59 cm^–1^, signature of SLG.^33^ The position of the G peak, Pos(G), is ∼1588 cm^–1^, with fwhm(G) ∼ 22 cm^–1^. The 2D peak position, Pos(2D), is ∼2679 cm^–1^, with fwhm(2D) ∼ 59 cm^–1^, while the 2D to G peak intensity and area ratios, I(2D)/I(G) and A(2D)/A(G), are ∼2.8 and ∼7.5. No D peak was observed, indicating negligible defects.^34^Figure 2 plots the Raman spectra of the paper-based PD at different fabrication stages. Raman spectra include the paper substrate (black), transferred graphene on a paper substrate before patterning (blue), and transferred graphene on a paper substrate after patterning and etching (green). In the Raman spectrum of paper substrate, as shown in Figure 2, the peaks ∼2850 cm^–1^ and ∼2890 cm^–1^ are attributed to the C–CH_3_ and aromatic C–H respectively, due to the resin coating on commercial glossy paper. For the transferred graphene on paper substrate, Pos(G) ∼ 1589.5 cm^–1^, fwhm(G) ∼ 30 cm^–1^, Pos(2D) ∼ 2695.4 cm^–1^, fwhm(2D) ∼ 34.9 cm^–1^, I(2D)/I(G) ∼ 3.8, and A(2D)/A(G) ∼ 4.5. Pos(2D) ∼ 2695.4 cm^–1^ indicates that layers were p doped.^35^ From A(2D)/A(G) ∼ 4.5 we estimate E_*F*_ ∼ 205 meV, by considering the dielectric constant ∼3^36^ of paper substrate. I(D)/I(G) ∼ 0.7 corresponds to a defect density ∼2.71 × 10^11^ cm^–2^^35^ for 2.41 eV excitation and E_*F*_ ∼ 205 meV. For transferred graphene on the paper substrate after patterning, etching, and PS removal, Pos(G) ∼ 1590 cm^–1^, fwhm(G) ∼ 27.8 cm^–1^, Pos(2D) ∼ 2695.9 cm^–1^, fwhm(2D) ∼ 38.1 cm^–1^, I(2D)/I(G) ∼ 4.5 and A(2D)/A(G) ∼ 3.6. Pos(2D) ∼ 2695.4 cm^–1^ indicates that layers were p doped.^35^ The Raman fitting of transferred graphene on a paper substrate, before and after patterning, is in agreement with the changes in doping levels, from a p-type doped E_*F*_ of 205 to 260 meV. This is consistent with changes in Pos(G), as well as decreases in fwhm(G) and A(2D)/A(G). From A(2D)/A(G) ∼ 3.6, we estimate E_*F*_ ∼ 260 meV, by considering the dielectric constant ∼3^36^ of paper substrate. I(D)/I(G) ∼ 0.63 corresponds to a defect density ∼2.8 × 10^11^ cm^–2^^35^ for 2.41 eV excitation and E_*F*_ ∼ 260 meV, thus no significant additional defects were induced during printed lithography.

**Figure 3:**
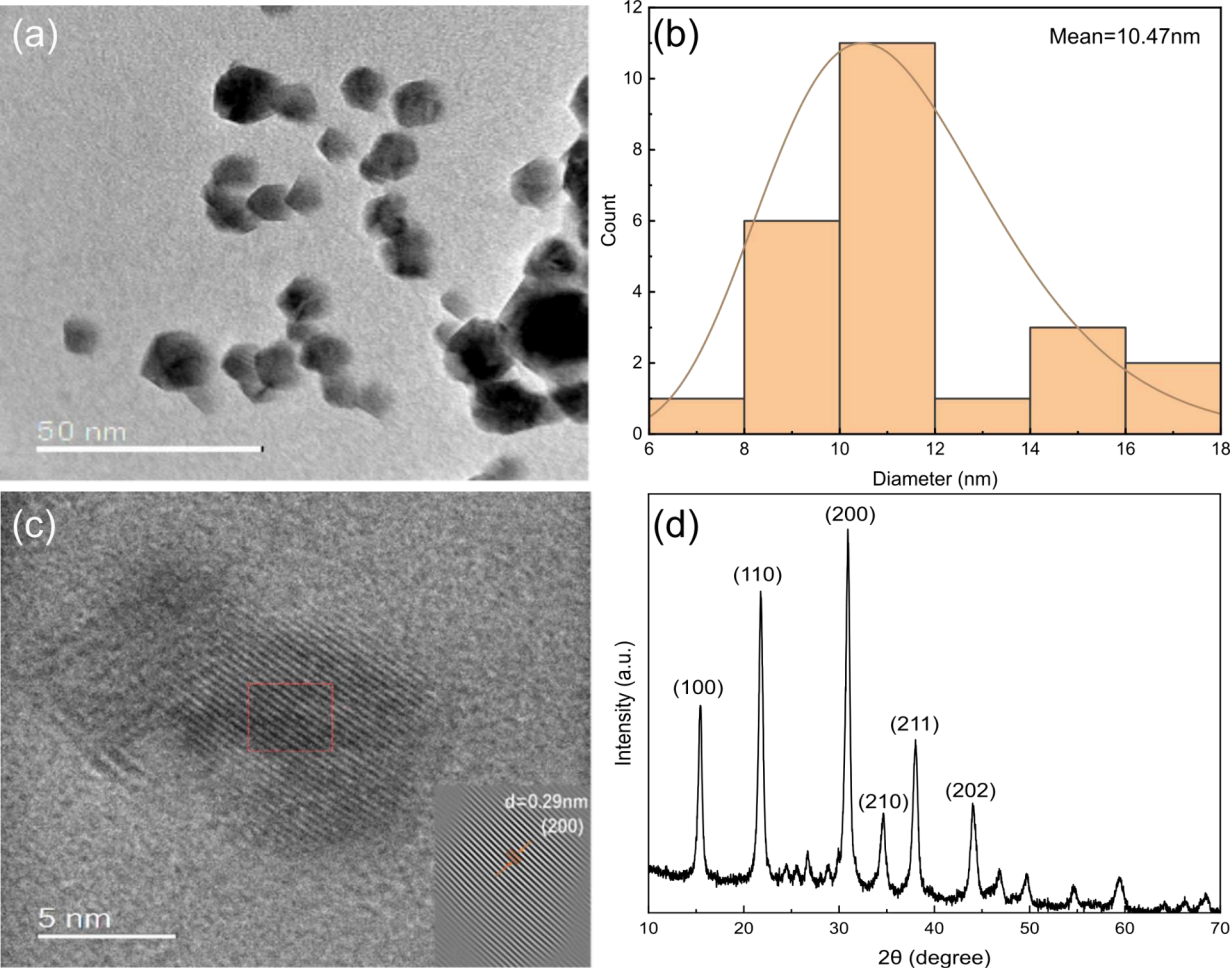
(a) TEM image of CsPbBr_3_ PQDs. (b) The corresponding size distribution spectra of CsPbBr_3_ PQDs. (c) High-resolution TEM image of CsPbBr_3_ PQDs showing the (200) crystal planes. The inset shows an enlarged high-resolution TEM image corresponding to the red rectangle. (d) XRD of the CsPbBr_3_ PQDs.

The synthesis of CsPbBr_3_ PQDs is detailed in the Materials and Methods section. CsPbBr_3_ PQDs were synthesized according to the method reported by Akkerman et al.^37^ To further explore the structural properties and assess the crystallinity of the synthesized CsPbBr_3_ PQDs, Transmission Electron Microscopy (TEM) analysis was conducted, as depicted in Figure 3. TEM images, shown in Figure 3a, illustrate the CsPbBr_3_ PQDs along with their size distribution. These PQDs tend to aggregate into larger clusters, suggesting that although the involvement of short-chain ligands aids in carrier transport, thereby enhancing the optoelectronic performance of the PQDs, it is ineffective in promoting uniform growth and dispersion of the PQDs. This result follows the same trend as that previously reported. The observed aggregates consist of crystalline domains approximately 10–20 nm in size, as inferred from Figure 3b. The size distribution analysis confirms an average particle size of approximately 10.47 nm, corroborating the observations from the TEM images and confirming the nanoscale dimensions of the synthesized PQDs. The reported average size of the PQDs is 15–20 nm in ref (37), which is slightly larger than our synthesized PQDs. This size discrepancy may be due to improvements in the washing method and adjustments in the amount of surface ligands, which led to a decrease in aggregation. Further structural details were obtained from high-resolution TEM images and XRD, presented in Figure 3c-d. The inset highlights the lattice fringes within the red rectangles corresponding to the (200) planes. The calculated lattice spacings are approximately 0.29 nm, which aligned well with the known orthorhombic phase of CsPbBr_3_ PQDs, confirming both the orthorhombic crystal structure and the high crystallinity of the synthesized samples. The crystallinity and crystal structure of the synthesized PQDs were also confirmed by the characteristic peaks of the XRD pattern (Figure 3d). Notably, six characteristic peaks are observed in the spectra, located at 15.2°, 21.6°, 31.9°, 34.8°, 37.9°, and 43.9°. According to the standard ICSD #14608, these peaks correspond to the lattice planes (100), (110), (200), (210), (211), and (202) of orthorhombic CsPbBr_3_ PQD crystals, respectively. In the XRD pattern, the height of the peaks is determined by the number of lattice planes arranged in the same direction. Notably, the peaks in the XRD pattern are narrow and sharp, indicating that the CsPbBr_3_ PQDs exhibit high crystallinity.

Atomic Force Microscopy (AFM) was also employed to assess the surface morphology and roughness of the fabricated PQDs. The surface exhibits a clear granular distribution with noticeable height differences between the grains, as shown in Figure S3a. To further quantitatively characterize the surface roughness, the root-mean-square (RMS) roughness value was measured as 4.9 nm, which is further explained in the Supporting Information. The scanning electron microscopy (SEM) cross-sectional image further visually demonstrates the layered structure of the device, as shown in Figure S3b. Measurements show that the thickness of the PQDs layer is approximately 13 μm. Figure S3c shows the thickness of the printed PQDs layer measured by the profilometer, which is about 12.6 μm, highly consistent with the SEM results. Considering the large carrier diffusion length of CsPbBr_3_ QDs,^38^ the printed PQDs achieve full surface coverage of the SLG channel, optimizing photodetection absorption even with the porosity of the printed layers.

**Figure 4:**
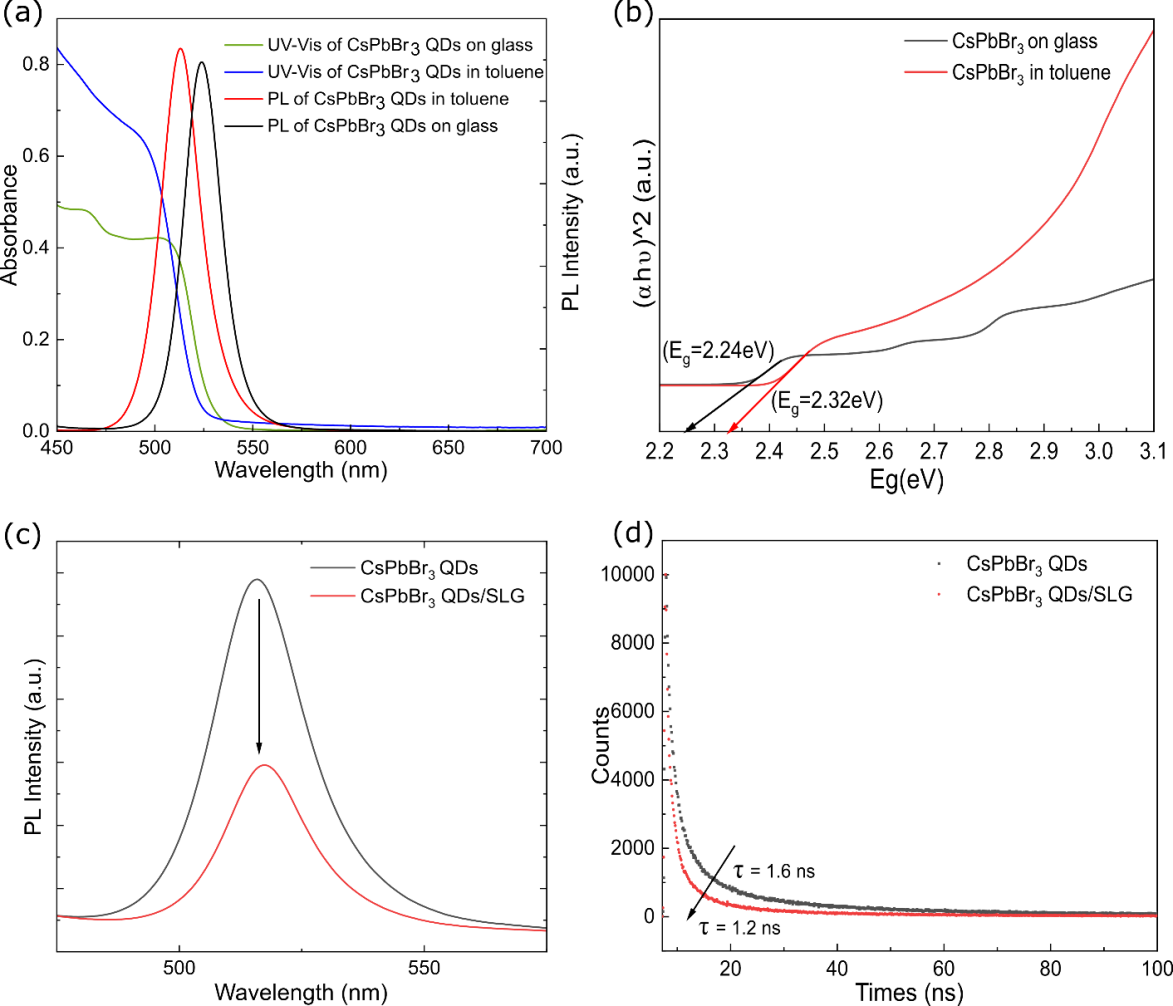
(a) UV–vis and PL spectra of CsPbBr_3_ PQDs on glass and dispersed in toluene. (b) Tauc plot of CsPbBr_3_ PQDs on glass and dispersed in toluene. (c) PL spectra of CsPbBr_3_ PQDs and CsPbBr_3_ PQDs/SLG on a glass substrate. (d) TRPL spectra of CsPbBr_3_ PQDs and CsPbBr_3_ PQDs/SLG on a glass substrate.

The photoluminescence (PL) and optical absorption spectra were obtained from CsPbBr_3_ PQDs that had been purified. This measurement was conducted for samples dispersed in toluene and microprinted on a glass substrate, as shown in Figure 4a. The PQDs in the solution exhibited a PL peak centered around 515 nm, with an fwhm of ∼21 nm, similar to the characteristics of cubic ∼9 nm nanocrystals,^39,40^ which is in close agreement with our statistical analysis from TEM images. Within the thin film, the optical absorption edge experienced a redshift of approximately 5 nm, transitioning from 515 to 520 nm, as determined from the band edge. This phenomenon could be attributed to surface defects, strain effects, and changes in size or shape.^41,42^ The PL observed from the film consistently tracked the absorption edge and maintained a consistent Stokes shift (approximately 6 nm) and fwhm like those observed in the solution. The Tauc plot method was applied to the UV–vis spectra to determine the bandgap of PQDs in different situation, 2.24 and 2.32 eV, respectively, as shown in Figure 4b. After microprinting and annealing, the crystal structure of PQDs grows larger, resulting in a red-shift of both absorption and emission peaks and also a reduction in the energy bandgap.

The PL intensity (integrated area under PL curve) of PQDs/SLG on glass was quenched ∼59% compared to PQDs on glass, as shown in Figure 4c. This can be assigned to charge carrier transfer between PQDs and SLG. To gain deeper insights into the PL reduction and charge transfer between PQDs and SLG, we employed time-correlated single photon counting (TCSPC) to investigate the excited-state dynamics of the PQDs/SLG superstructure. Figure 4d shows the time-resolved photoluminescence (TRPL) spectra of CsPbBr_3_ PQDs with and without SLG on glass substrates. It displayed a biexponential decay pattern similar to what has been previously reported in the literature,^43^ with an average fluorescence decay time of 1.6 ns, the PQDs/SLG exhibited a notably shorter average fluorescence decay time of 1.2 ns, as shown in Figure 4d. From the collected data, SLG demonstrated similar quenching effects to those reported for PQDs deposited on SLG.^44^ The shortened fluorescence lifetimes observed in this investigation, coupled with the previously reported low exciton binding energy of perovskite (PVK) materials, may suggest a dominant photoinduced electron transfer mechanism contributing to the quenching effects. The presence of PQDs on SLG can enhance charge transfer by enabling π–π electron interactions between PQDs and the sp^2^-hybridized graphene layer.^45^ Therefore, the Pb and Br orbitals in CsPbBr_3_ PQDs can overlap with the unhybridized 2p orbitals of the carbon atoms in graphene, and the efficacy of charge transfer at the interface of PQDs and graphene depends on the overlap of the π orbitals. In essence, the observed TRPL quenching signifies rapid charge transfer within the PQDs/SLG superstructure, facilitated by suitable band alignment and the interaction at the interface between the PQDs and SLG.

**Figure 5:**
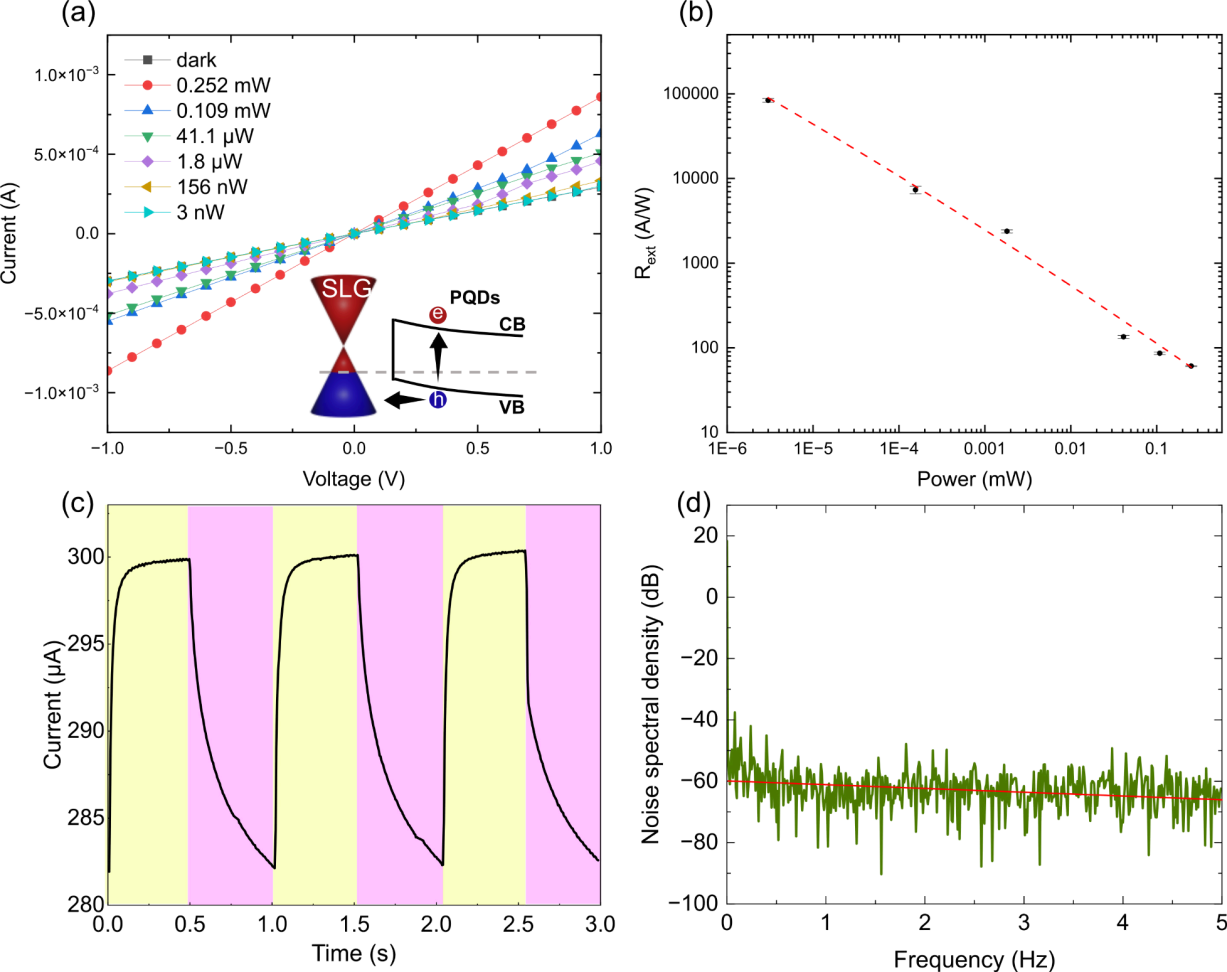
Performance of the PD. (a) I–V curves of the PD irradiated by a 520 nm laser, with different input powers. Inset shows a schematic band diagram of the PQDs/SLG interface. (b) R_*ext*_ of the PD as a function of the optical power. Data are presented as mean ± SD (*n* = 3). (c) Temporal photocurrent response under alternating dark (purple) and light (yellow) conditions. (d) Spectral noise density of paper-based PDs.

The performance tests of PDs are shown in Figure 5. The operating principle of our PDs is demonstrated in Figure 5a. For energy band alignment, the Dirac point of SLG is assumed to be ∼0.26 eV, and SLG is considered p-type doped based on our Raman measurements. A schematic band diagram of the SLG/PQDs interface is shown in Figure 5a, which indicates the PQDs conduction band (CB) and valence band (VB), generation of electron–hole (e/h) pairs, and transfer of holes (h) from PQDs to SLG. Upon illumination and photon absorption in PQDs, electron–hole pairs are generated in the PQDs. Part of the photogenerated holes would be injected from the PQDs valence band into the lower energy states in p-doped SLG, leaving behind the uncompensated charge of photogenerated electrons. These electrons act as an additional negative gate bias, resulting in a photogating effect.^46^ At the SLG/PQDs interface, a built-in electric field is formed, causing holes to transfer to the SLG under the influence of this field, while the electrons remain trapped in the PQDs. The latter would induce a stronger electric field at the SLG/PQDs interface, thus favoring holes transfer from PQDs. The injected holes from the PQDs occupy energy states above E_*F*_, increasing both the hole concentration and the hole current in the SLG channel. Figure 5a shows a series of I–V curves of the PD under irradiation with different input powers of the 520 nm laser. The linear relationship between current and voltage indicates Ohmic contact between CVD SLG and Ag electrodes. The photocurrent is defined as
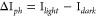
1where I_*light*_ represents the current produced by the device when exposed to light, and I_*dark*_ is the current measured in the absence of light. We conducted all measurements at a bias voltage of 1 V to ensure that the device operated within its linear (Ohmic) operating range. For V_*ds*_ > 1 V, the free carriers drift velocity *v*_*d*_=μE/(1+μE/*v*_*sat*_),^47^ with *v*_*sat*_ the saturation velocity of the carriers in the SLG channel, and *E* the applied electric field to SLG, increases linearly, until saturation, due to carrier scattering with optical phonons.^48^ Therefore, all measurements are done at V_*ds*_ ≤ 1 V to keep the device operation in the linear (Ohmic) regime, thus eliminating the nonlinear dependence of *v*_*d*_ on V_*ds*_.

The photocurrent of the PD has a significant dependence on the light intensity. An increase in the incident power (P_*opt*_) results in higher photocurrents (0.435 mA at 1 V), primarily because of the elevated rate of recombination among photoexcited carriers.

Responsivity can be expressed as external and internal. Internal responsivity estimates photodetection efficiency, characterizing the photoconversion process of absorbed photons.^49^ On the other hand, the R_*ext*_ describes the overall PD responsivity. R_*ext*_ accounts for factors such as PD design and architecture, light absorption and reabsorption, optical reflection at the interface, the optical path in the photoactive material, and material quality.^50^ R_*ext*_ is given by the following equation:
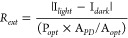
2where I_*light*_ and I_*dark*_ are the currents of the PD under 1 V bias voltage in illumination and in dark conditions. A_*PD*_ and A_*opt*_ are the PD area (7.31 × 10^–7^ m^2^, 884.6 μm in length, 826.9 μm in width) and the beam size (1.96 × 10^–5^ m^2^). A_*PD*_/A_*opt*_ is a scaling factor that considers the fact that only a fraction of the optical power impinges on the PD. P_*opt*_ is the incident optical power. To derive R_*ext*_, we measure Δ*I*_*ph*_ at different optical powers via an attenuator ranging from 252 μW to 3 nW. As shown in Figure 5b, at a bias of 1 V, R_*ext*_ increased from ∼60 AW^–1^ to ∼82,300 AW^–1^ when the input power of the 520 nm laser decreased from 252 μW to 3 nW. This change in R_*ext*_ can be explained by the shielding of the built-in electric field. When light is illuminated into the photoactive layer of the device, the generated electron–hole pairs are separated by the built-in electric field created at the interface between the SLG and PQDs, causing holes to transfer into the SLG and increase its hole concentration, resulting in increased hole conductivity in the SLG. At high light intensities, more electrons at the PQDs/SLG interface recombine with h, resulting in a lower R_*ext*_. However, as the light intensity decreases, fewer electrons at the interface are recombined, reflecting a higher R_*ext*_.^46,49^ To account for the light response of the paper substrate, we tested the photoresponse of the bare paper, as shown in Figure S4–5. We observed no significant changes under light and dark conditions, indicating that the paper substrate shows no responsivity. This finding is consistent with other reported literature, which also notes negligible changes in the photocurrent of the paper substrate.^51^

External quantum efficiency (EQE), defined as the number of electrons detected per incident photon, is another key parameter for PD. The larger the EQE, the higher the sensitivity of the PD. EQE is given by the following equation:^52^
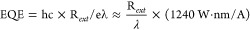
3where λ is the wavelength of the incident light in nm, h is the Planck constant, c is the speed of light in vacuum, and e is the elementary charge. The EQE of our PQDs/SLG PDs is shown in Figure S6. The EQE reached ∼ 1.96 × 10^7^ %, which follows a similar pattern to R_*ext*_ on a function of optical power.

The temporal response of our PDs is then measured at 520 nm, Figure 5c. Rise time (τ_*rise*_) and fall time (τ_*fall*_) were calculated based on the time it takes for a signal to go from 10% to 90% of the photocurrent for the τ_*rise*_, and from 90% to 10% of the photocurrent for the τ_*fall*_. We get a τ_*rise*_ ∼ 51 ms and τ_*fall*_ ∼ 338 ms, faster than 470 ms of Bi_2_S_3_/MoS_2_ PDs on paper substrate,^8^ and PbS paper-based PDs, with response and recovery times of 360 and 410 ms, respectively.^53^ The response time of graphene-PQD PDs is influenced by several factors including carrier lifetime, recombination rates, trap states, interface quality, and substrate choice. PQDs generally suffer from slower carrier transport and recombination compared with graphene, which hinders their response speed. Effective charge transfer between the PQD and graphene can mitigate this, but the recombination rate in the PQD layer is still a limiting factor.^54^ Moreover, the interface quality between the graphene and PQD layers is a critical factor. Imperfections at the interface can lead to inefficient carrier separation and slower charge transport, which negatively impact the device’s response time. By improving the charge transport layers (e.g., modulating surface traps or interface engineering), the response speed can be significantly enhanced.^55,56^ Additionally, reducing the thickness of the PQD layer and improving the electrical properties of the contact layers can reduce the response time.^57^

To evaluate the detection of PD, normalized detectivity (D* (cm· Hz^1/2^/W or Jones)) is calculated. D* relates the performance of PDs in terms of R_*ext*_ to the photoactive area of the PD, allowing for the comparison of PDs with different active areas.
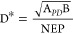
4where B is the electrical bandwidth (Hz) and NEP is the noise equivalent power (i.e., the power that gives a signal-to-noise ratio of one in a 1 Hz output bandwidth.
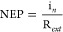
5where i_*n*_ is the dark noise current, i.e., the current that exists when no light is incident on the PD. The noise (A/Hz) is measured in the time domain by collecting the trace on an oscilloscope, followed by a Fourier transform to analyze the data in the spectral domain. Figure 5d plots the 1/f noise (where f is the frequency). 1/f is the noise density (noise power per unit of bandwidth (dBm· Hz), due to charge traps and defects in the material.^58^ Subsequently, we get NEP ∼ 1.1 × 10^–11^ W/√Hz and D* ∼ 7.8 × 10^9^ Jones, which is higher than printed hybrid CVD SLG and black phosphorus PDs on Si/SiO_2_ substrate with D* 2 × 10^7^ Jones.^49^ Thus, our printed PDs are suitable for detecting weak light intensities which compete with the detector noise.^58^ The noise current in the shot noise limit is given by^59^
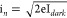
6where e is the elementary charge (1.6 × 10^–19^ C) and I_*dark*_ is the total dark current combines the contribution of the unamplified SLG current and the injection current from PQDs, due to the thermal excitation of charge carriers in dark. The latter was orders of magnitude smaller compared to the dark current from SLG ∼ 293 μA. Therefore, following the methodology presented in Bianconi et al.,^60^ in our devices the shot noise-limited current was primarily due to the SLG layers. For the specific case of a bandwidth of 1 Hz, D* based on shot noise limit is calculated as ∼7.3 × 10^10^ cm· Hz^1/2^/W in our paper-based PQDs/SLG PDs which is quite enhanced compared to paper-based PDs functionalized by WS_2_ nanosheet/graphene,^61^ WSe_2_ nanosheets/PANI,^62^ and WSe_2_/CuO^63^ reported previously.

**Figure 6:**
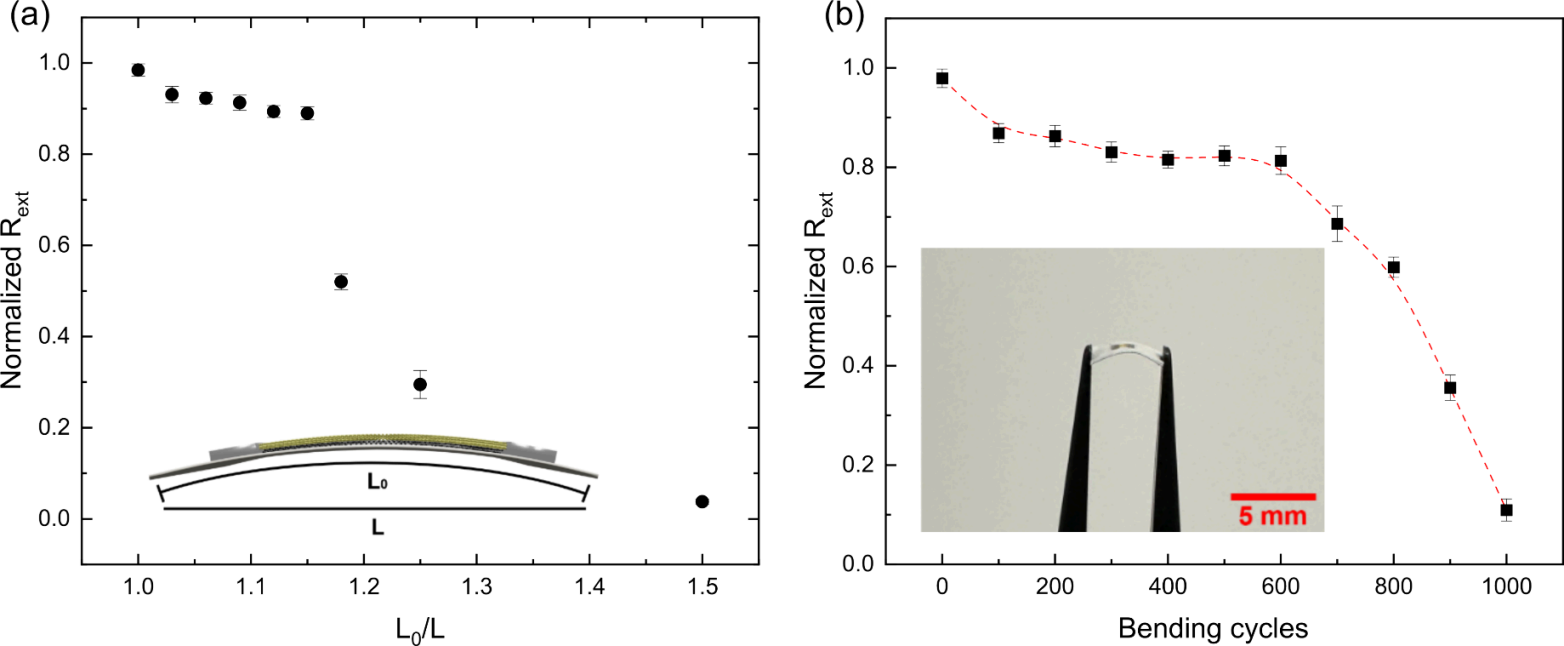
(a) Normalized R_*ext*_ of PD at different bending angles under illumination of 0.2 mW input power. Inset shows a schematic diagram of the PD in a bent state. (b) Normalized R_*ext*_ of PD at a 1.15 bending angle and different bending cycles, under the illumination of 0.2 mW input power. Inset shows an optical image of the PD in the bent condition (L_0_/L = 1.15). Data are presented as mean ± SD (*n* = 3).

Benefiting from the unique flexibility of paper, the PDs prepared in this project maintained their superior performance even in the bending state. To test the performance of PD in the bending state, the ratio of arc length (L_0_) and chord length (L) (L_0_/L) was used to define the degree of bending of PD (inset image of Figure 6a).^51^ The surface morphology of the paper substrate before and after the flexibility test is shown in Figure S7 and S8. When the incident laser power at 520 nm was fixed at 0.2 mW, the I–V curves were shown in Figure S9, and the corresponding responsivity of the PD at different bending angles was shown in Figure 6a. It can be seen that the PD’s R_*ext*_ decreases slightly in the initial bending. Afterward, the R_*ext*_ remained almost constant during subsequent bending until L_0_/L = 1.18, at which point it showed a sharp decrease. This behavior has also been observed in other studies.^64^ This could be attributed to the construction of some new conductive networks and the subsequent formation of an equilibrium state. When the bending degree exceeds L_0_/L = 1.15 (bending radius: 2.8 mm), the PD experiences structural damage, leading to a sharp decline in R_*ext*_ at 4% at L_0_/L = 1.5. Beyond this point, the device becomes inoperative.

Figure S7a-b and Figure S8a-b show the three-dimensional mapping and SEM images of the paper substrate before and after bending at L_0_/L = 1.18, respectively. It can be seen that there was no significant damage to the surface of the paper substrate. This observation, in conjunction with the pronounced reduction in the R_*ext*_ of the PD in Figure 6a at L_0_/L = 1.18, leads to the inference that the observed deterioration in performance was likely attributable to structural damage within the PD. To test the durability of the PDs, we tested the performance of the PDs under different bending cycles, the responsivity as shown in Figure 6b and corresponding I–V curves were shown in Figure S10. After 600 bending cycles, the PD still retained 80% responsivity, indicating that the PD exhibits excellent durability. After the 700th bending, the responsivity sharply decreased to 73%, which is still much higher than other paper-based PDs (shown in Table 1). Figure S7c-d and Figure S8c-d show the three-dimensional mapping and the SEM images of the paper substrate subsequent to 600 and 1000 bending cycles under the condition of L_0_/L = 1.15, respectively. Wrinkles form on the surface of the paper substrate after 600 cycles, while breakage occurs on the surface after 1000 cycles. This observation is consistent with the data presented in Figure 6b, which shows that the performance of the PD remains approximately at 80% after 600 bending cycles at L_0_/L = 1.15. Subsequently, a sharp reduction in the R_*ext*_ of the PD to around 10% is observed, which is considered to be partially due to the surface damage of the paper substrate. For the reproducibility of our devices, we fabricated and tested six additional PDs. The results show consistent performance across all devices, which is further explained in Supporting Information (Figure S11). As shown in Figure S12, after a five-week storage period at room temperature, the R_*ext*_ of the CsPbBr_3_ PQDs/graphene PD retained more than 90% of its initial R_*ext*_. The stability of our PDs is attributed to the high stability of CsPbBr_3_ PQDs, which serve as the main photoabsorbent layer, consistent with the high stability reported for these PQDs.^65^

The current state-of-the-art PDs based on paper or 2d materials are summarized in Table 1. Fang et al. developed a flexible PD by exploiting the rough and porous nature of ordinary printing paper; they drew electrodes using a pencil and then drop-cast CH_3_NH_3_PbI_3_ ink between the electrodes as the active region.^69^ Although this PD retained 91.23% of its photocurrent after 1,000 bending cycles (bending radius of 14 mm), it exhibited a low responsivity of only 4.4 × 10^–3^ AW^–1^ at a bias voltage of 5 V.^69^ Deng et al. fabricated a flexible PD based on CsPbBr_3_/Ti_3_C_2_T_*x*_ through spray coating, achieving a responsivity of 4.49 × 10^–2^ AW^–1^ at 10 V, with the photocurrent maintaining 85% after 1,500 bending cycles.^70^ Li et al. used drop coating to fabricate a flexible PD based on MAPbBr_3_ on filter paper, which demonstrated a responsivity of 1.3 AW^–1^ under a low bias voltage of 1 V, and retained 85.6% of its photocurrent after 1,000 bending cycles.^51^ Malik et al. reported a paper-based PD using spray coating, achieving the highest responsivity of 520 AW^–1^ at 1 V; however, the device exhibited approximately 85% degradation after 500 bending cycles.^72^ Among these PDs, the one developed by Li et al. exhibited the high responsivity and the lowest bias voltage, along with excellent bending performance compared to other studies.^51,69,70,72^ However, its responsivity is 4 orders of magnitude lower than the value we report. To the best of our knowledge, our PQD/SLG PD exhibits the highest R_*ext*_ among the paper-based PDs, as shown in Table 1. The R_*ext*_ can be further enhanced by applying a higher source-drain voltage bias.

## Conclusion

We have demonstrated high-performance paper-based PDs made with CVD SLG and CsPbBr_3_ PQDs using a microplotter under ambient conditions. We achieved an R_*ext*_ of approximately 82,000 AW^–1^ at 520 nm with an operating voltage of 1 V. Our fabrication method is ideal for producing scalable electronics on paper. After 600 bending cycles at L_0_/L = 1.15, the PD retained up to 80% of its R_*ext*_. This research highlights the immense promise of creating active devices for future paper-based electronics, paving the way for cost-effective, eco-friendly, and sustainable real-world applications.

## Materials and Methods

### Materials

SLG on Cu was purchased from Graphenea. Glossy paper was purchased from Canon (Canon GP-501). PMMA (molecular weight: 120,000 g/mol), anisole (CH_3_OC_6_H_5_), APS (NH_4_)_2_S_2_O_8_), PS (C_8_H_8_)_*n*_, (molecular weight: 192,000 g/mol), toluene (C_6_H_5_CH_3_), lead(II) bromide (PbBr_2_, 99.99%), cesium, carbonate (Cs_2_CO_3_, 99%), butylamine (BuAm, 99.5%), 2-propanol (IPrOH, anhydrous, 99.5%), propionic acid (PrAc, ≥ 99.5%), *n*-hexane (HEX, 99.5%), toluene (TOL, anhydrous, 99.8%), and Ag nanoparticles dispersion were purchased from Sigma-Aldrich (Ag dispersion, 736465). Unless otherwise stated, all chemicals were of analytical grade

### Synthesis of CsPbBr_3_ PQDs

**Figure 7:**
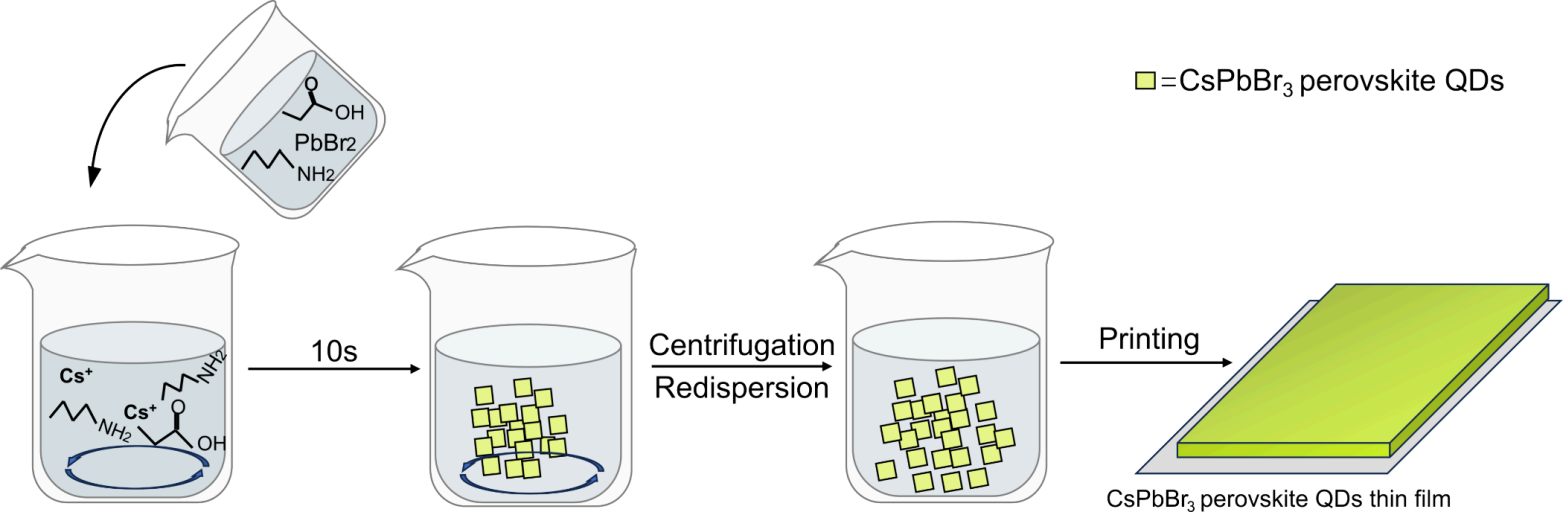
Schematic of the synthesis process of CsPbBr_3_ PQDs.^37^

The synthesis process for the CsPbBr_3_ PQDs, as illustrated in Figure 7, is a straightforward and rapid one-step injection method. The process commences by utilizing PrAc to dissolve Cs_2_CO_3_, creating a Cs^+^ propionate complex that was then diluted within a mixture of polar and apolar solvents comprising isopropyl alcohol (IPrOH), hexane (HEX), and butylamine (BuAm) at room temperature. This dissolution reaction is exothermic, obviating the need for an external heating. Notably, unlike prior room-temperature synthesis techniques, there is no requirement for degassing the precursors, and the entire procedure takes place under a nitrogen atmosphere to prevent unwanted reactions. Simultaneously, a distinct solution was prepared under ambient conditions, involving the dissolution of PbBr_2_ within a similar chemical mixture (also at room temperature). This solution was then injected into the first mixture. Within a mere 10 s postinjection, the PQDs initiate nucleation and rapidly reach their maximum size. The synthesis of CsPbBr_3_ PQDs often involves a visible color change reaction. Typically, within 10 s of the (Cs^+^ and PbBr_2_ precursors) coming into contact, the reaction starts, leading to the gradual emergence of a fluorescent yellow solution. In as little as two minutes, the reaction reaches its end point, with no further product formation. After this stage, the PQDs were isolated through centrifugation and redispersed in toluene, rendering them ready for direct utilization in device fabrication. The dimensions of the PQDs were adjustable through modulation of the IPrOH to HEX ratio. An escalation in this ratio, thereby augmenting solution polarity, results in larger crystalline domains. However, this approach yields unstable colloidal solutions, preventing further exploration of this method.

### Device Fabrication

To transfer graphene, PMMA solution diluted in anisole (10 wt %) was spin-coated at 3000 rpm for 20 s on the graphene-grown Cu foil. Backside graphene on the Cu foil was removed by O_2_ plasma (Diener electronic GmbH & Co.) treatment for 20 s at 30 W radio frequency power. Then the sample was placed in a 65 mM APS solution overnight, whereby Cu was chemically etched. The glossy paper substrate was first cleaned by acetone, IPA, and DI water, and dried using flowing nitrogen gas. The PMMA/SLG was then moved to a beaker with DI water to remove APS residuals and lifted with the target glossy paper substrate. After drying, PMMA was removed in acetone and IPA, leaving SLG on the substrate. Single and double-layer graphene samples were prepared according to the above method. The PS solid was added to toluene to make a 5 wt % solution, then drawn on graphene. The drawing process was accompanied by using a Microplotter Proto (Sonoplot, UK) equipped with a 10 μm diameter piezoelectric driven nozzle. After printing, the graphene with PS ink was patterned using an O_2_ plasma. Then, the PS was removed in acetone and IPA. After drying, the Ag nanoparticle dispersion was printed on each end of the patterned graphene using Microplotter Proto to form electrodes. The devices were annealed in air at 150 °C for 2 h to improve the contact of the graphene and electrodes. The PQDs were printed on graphene and then dried at 80 °C for 1 min. This process was repeated 5 times to ensure full contact of the PQDs and complete coverage of the targeted area.

### Characterization

Optical absorption spectra were measured using Lambda 750S UV–vis spectrometers (PerkinElmer) at room temperature. Steady-state and time-resolved PL spectra were obtained on a TCSPC (LifeSpec-ps) from Edinburgh Instruments with an excitation wavelength of 405 nm. Optical photographs of PD were taken using an Axio optical microscope (ZEISS). Raman Spectra were measured by inVia Raman microscope (Renishaw) at room temperature with an excitation wavelength of 515 nm and 100× objective. Performance tests of PD were obtained on a B1500A Semiconductor Device Analyzer (Keysight) The time response signal was collected with a high-sensitivity oscilloscope (4 GSa/s real-time sample rate with a high sensitivity of 100 μV) with an input impedance of 1 MΩ. The sheet resistance of different layers of CVD graphene was measured by a Four-Point Probe (Ossila). AFM was performed using Nanosurf CoreAFM. Contact angle and surface tension measurements were performed using Theta Optical Tensiometers (Biolin Scientific) in Sessile drop mode. The SEM characterizations and profilometer measurements were performed using SEM (Zeiss) and stylus profilometer (Bruker).

## Data Availability

All data are available within the article or available from the authors upon reasonable request.

## Supporting Information Available

Additional experimental details, materials, and methods, including CVD-wet transfer, surface tensions, contact angles, atomic force microscopy images, scanning electron microscopy images, profilometer, photographs of printed photodetectors on papers, the response time of photodetectors, calculated external quantum efficiency, responsivity, and stability measurements of photodetectors (PDF)
