# Supplementary material for: Printed Lithography of Graphene-Perovskite Quantum Dot Hybrid Photodetectors on Paper Substrates
Source: ACS Appl Mater Interfaces. 2025 Jan 20;17(4):6716–27. doi: 10.1021/acsami.4c18102 (PMC11788987; doi:10.1021/acsami.4c18102)
Supplement: Supplementary file 1 — am4c18102_si_001.pdf [file am4c18102_si_001.pdf]

Supporting Information
Printed Lithography of Graphene-Perovskite Quantum Dot Hybrid Photodetectors on Paper Substrates

Yujia Li,[†,‡,§] Yining Zhao,[†,‡,§] Alfonso Ruocco,[¶] Mingqing Wang,[†] Bing Li,[*,†] and Shahab Akhavan[*,†]

†*Institute for Materials Discovery, University College London, London, WC1E 7JE, UK*

‡*Department of Chemistry, University College London, London, WC1E 7JE, UK*

¶*Optical Networks Group, University College London, London, WC1E 6BT UK, UK*

§*These authors contributed equally to this work*

E-mail: bing.li@ucl.ac.uk; s.akhavan@ucl.ac.uk

To transfer graphene (Graphenea, Inc., USA), polymethyl methacrylate (PMMA) solution diluted in anisole (10 wt%) was spin-coated (Ossila, UK) at 3000 rpm for 20 s on the graphene-grown Cu foil. The backside graphene on Cu foil was removed by O_2 plasma (Diener electronic GmbH & Co., UK) treatment for 20 s at a radio frequency power of 30 W. Then the sample was placed in a 65 mM ammonium persulfate (APS) solution overnight, whereby Cu is chemically etched. The glossy paper substrate was first cleaned with acetone, isopropyl alcohol (IPA), and DI water, after being dried by using flowing nitrogen gas. The PMMA/graphene was then moved to a beaker with DI water to remove APS residuals and lifted with the target glossy paper substrate. After drying, PMMA was removed in acetone and IPA, leaving graphene on the substrate. For bilayer graphene, the above process was repeated.

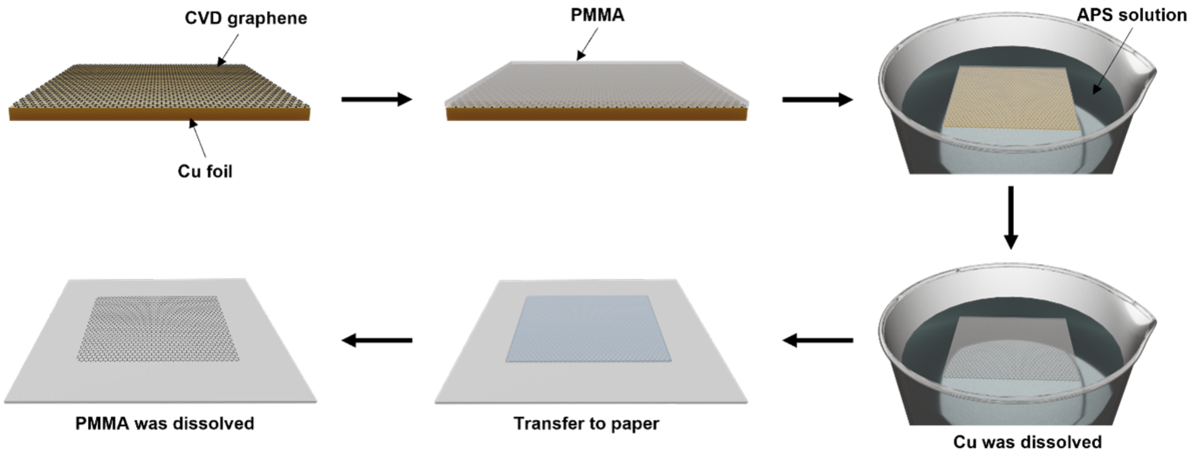

Figure S1: Schematic of wet-transfer CVD graphene process.

Table S1: Sheet resistance of different layers of CVD graphene via wet-transfer (Data are presented as mean±SD, n=11).

Layer	Sheet resistance (Ohm/square)
1	5246.00±5.13
2	2255.65±2.59
3	2290.39±0.88
4	2486.61±4.30

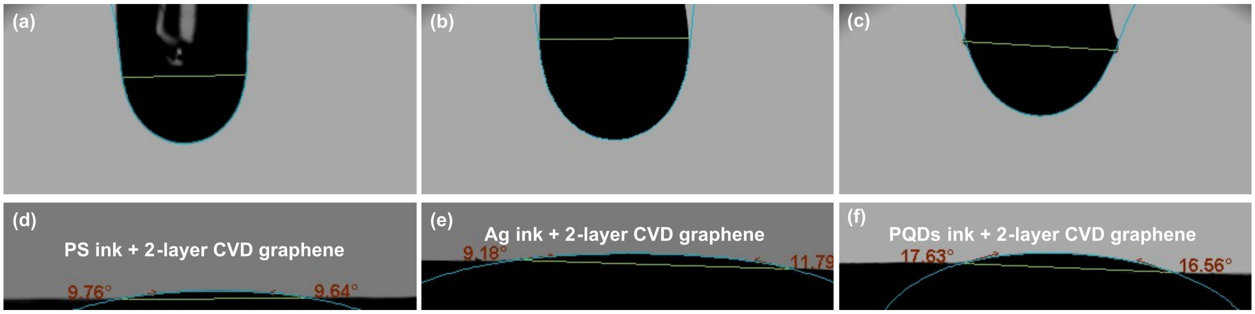

Figure S2: Surface tension of (a) polystyrene (PS) ink, (b) silver ink, (c) $CsPbBr_3$ PQDs ink. Contact angle of (d) PS ink on bilayer CVD graphene, (e) silver ink on bilayer CVD graphene, (f) $CsPbBr_3$ PQDs ink on bilayer CVD graphene.

Table S2: Surface tension of PS ink, silver ink, and $CsPbBr_3$ PQDs ink (Data are presented as mean±SD, n=11).

Inks	Surface tension (mN/m)
PS ink	2.18±0.13
Silver ink	2.78±0.03
$CsPbBr_3$ PQDs ink	1.82±0.08

Atomic Force Microscopy (AFM) was also employed to assess the surface morphology and roughness of fabricated PQDs. The surface exhibits a clear granular distribution with noticeable height differences between the grains, as shown in Fig. S3(a). Based on the image scale (both horizontal and vertical axes are in micrometers), the lateral size of the grains is approximately 0.1 to 1 μm. This result is significantly larger than the crystal size of the PQDs, which may be due to the aggregation of crystal particles during the spin-coating process. The color bar on the right represents the potential distribution (in volts), ranging from -0.002 V to 0.449 V. According to the color distribution in the image, there are potential differences in different regions of the surface. In some areas, large potential differences can be observed, which may be related to the crystal orientation or local defects. Regions with higher potential values (close to 0.4-0.449 V) appear bright white, while regions with lower potential values (close to 0.0 V) appear dark, Fig. S3(a). This suggests that there may be a high local charge accumulation in these bright white areas, which could be due to a more ordered crystal arrangement and lower defect density in these regions. To further quantitatively characterize the surface roughness, the root mean square (RMS) roughness value was measured as 4.9 nm, according to the $RMS(nm){=}RMS(V) \times Calculated\ Deflection\ Sensitivity\ (nm/V)$ where calculated deflection sensitivity can be found during the calibration process before the measurement. (here is 45.2 nm/V for the adopted tip).

The SEM cross-sectional image further visually demonstrates the layered structure of the device, Fig. S3(b). Bilayer graphen were transferred onto the glossy paper substrate, followed by the printing of the PQDs absorption layer. On the left side, the printed silver electrode by the microplotter can be clearly seen. Measurements show that the thickness of the QDs layer is approximately 13 μm. The Fig. S3(c) shows the thickness of the printed PQDs layer measured by the profilometer, which is about 12.6 μm, highly consistent with the SEM results.

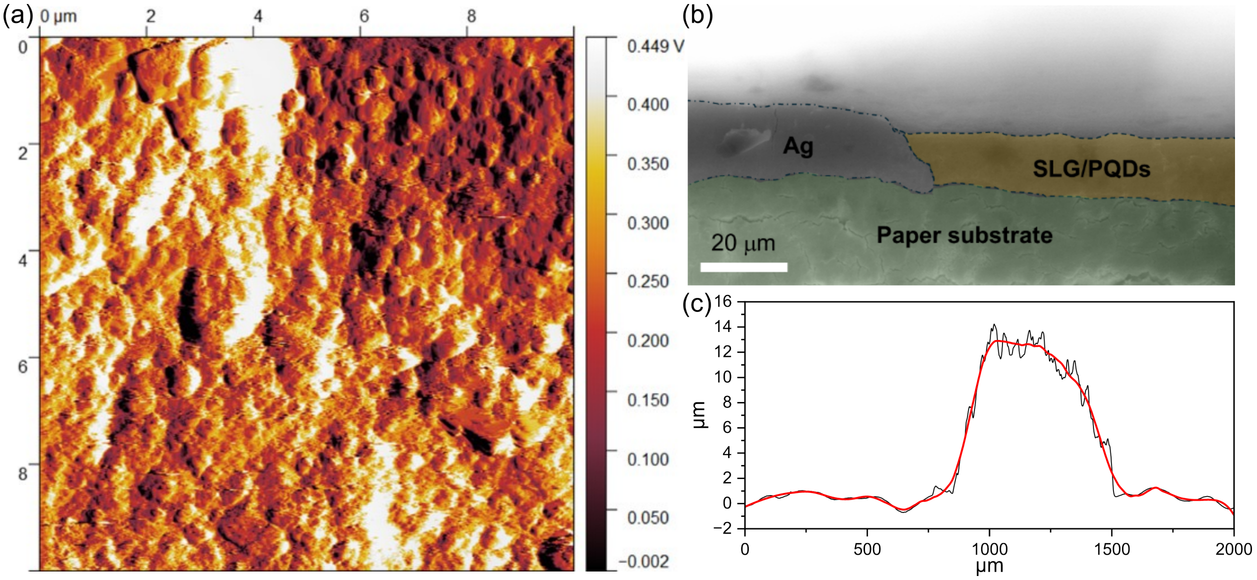

Figure S3: (a) AFM image of $CsPbBr_3$ PQDs. (b) SEM cross-section image of the fabricated device. (c) Thickness measurement of $CsPbBr_3$ PQDs layer via profilometer.

To measure the light response of the paper and determine whether the bare paper contributes to overall responsivity, we tested the responsivity of the bare paper, as shown in Fig. S4 and S5. We observed no significant changes under light and dark conditions, indicating that the paper substrate exhibits no responsivity. This finding is consistent with other reported literature, which also notes negligible changes in the photocurrent of the paper substrate.[1]

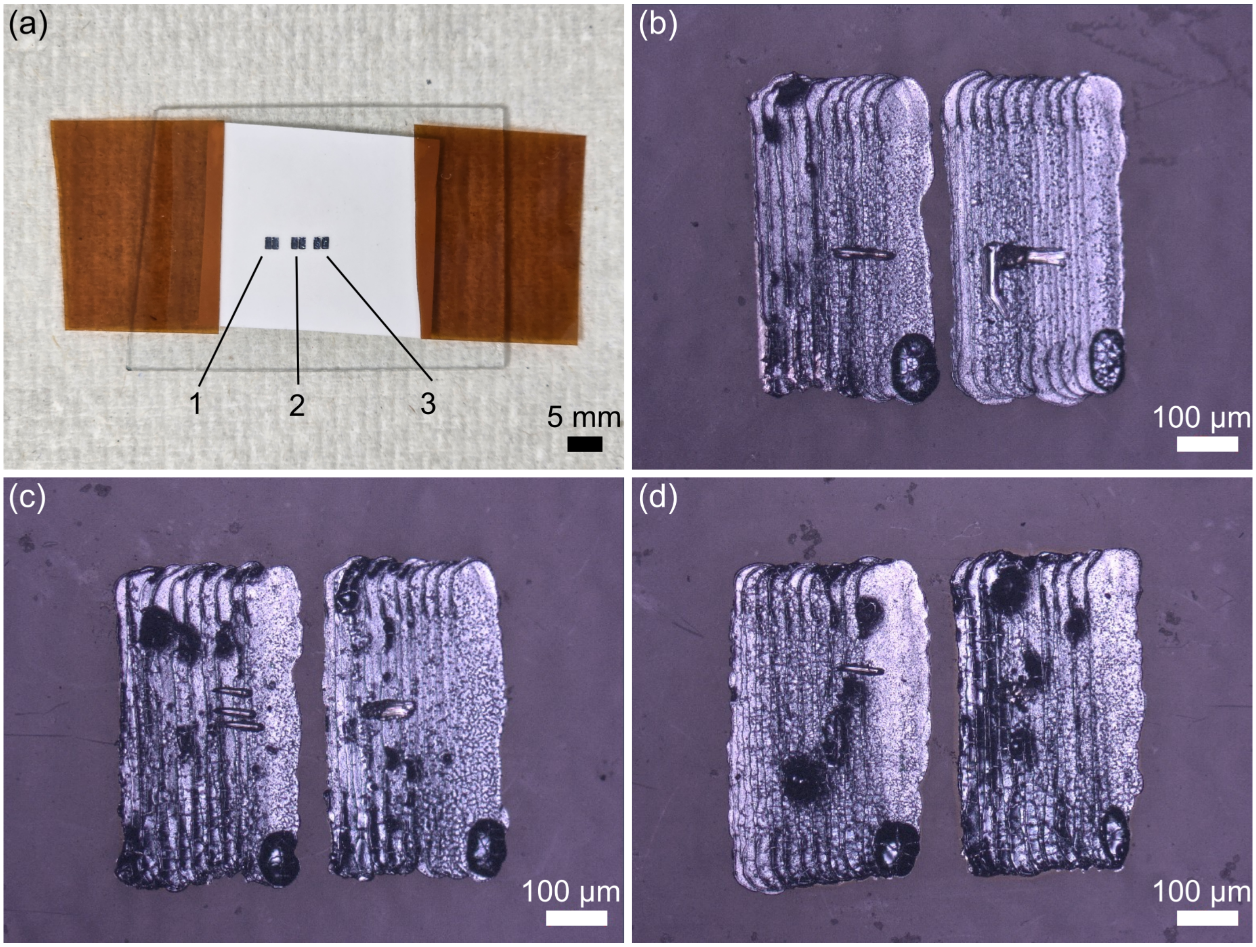

Figure S4: Three printed pairs of silver electrodes, with a distance of ∼40 μm between each electrode (identical to the channel sizes for the proposed PDs on a bare paper substrate), were used to characterize the response of the bare paper substrate to light and its possible contribution to the overall responsivity of the PDs. (a) Photo of printed silver electrodes on paper substrate; (b-d) Optical microscope images of silver electrodes 1 to 3 (slight scratches on the printed silver electrode surface are due to marks left by the probes during measurements).

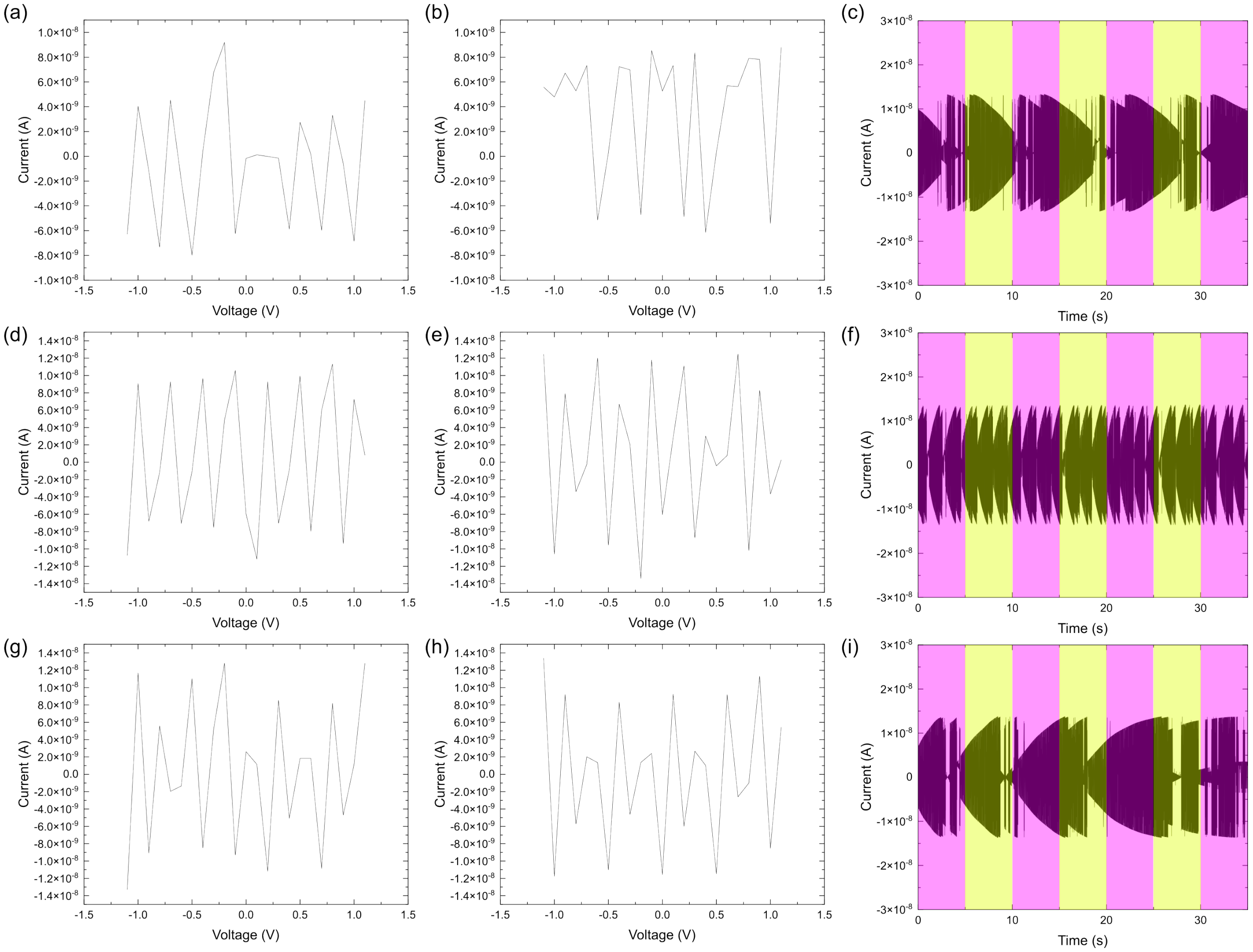

Figure S5: The response of the paper substrate to light (at 520 nm and 0.2 mW) was measured for three pairs of printed silver electrodes (1, 2, and 3) on the paper substrate, with ∼40 μm between them. (a) I-V curve of silver electrodes 1 in dark conditions; (b) I-V curve of silver electrodes 1 irradiated by a 520 nm laser; (c) Temporal photocurrent response of silver electrodes 1 under alternating dark (purple) and light (yellow); (d) I-V curve of silver electrodes 2 in dark conditions; (e) I-V curve of silver electrodes 2 irradiated by a 520 nm laser; (f) Temporal photocurrent response of silver electrodes 2 under alternating dark (purple) and light (yellow); (g) I-V curve of silver electrodes 3 in dark conditions; (h) I-V curve of silver electrodes 3 irradiated by a 520 nm laser; (i) Temporal photocurrent response of silver electrodes 3 under alternating dark (purple) and light (yellow).

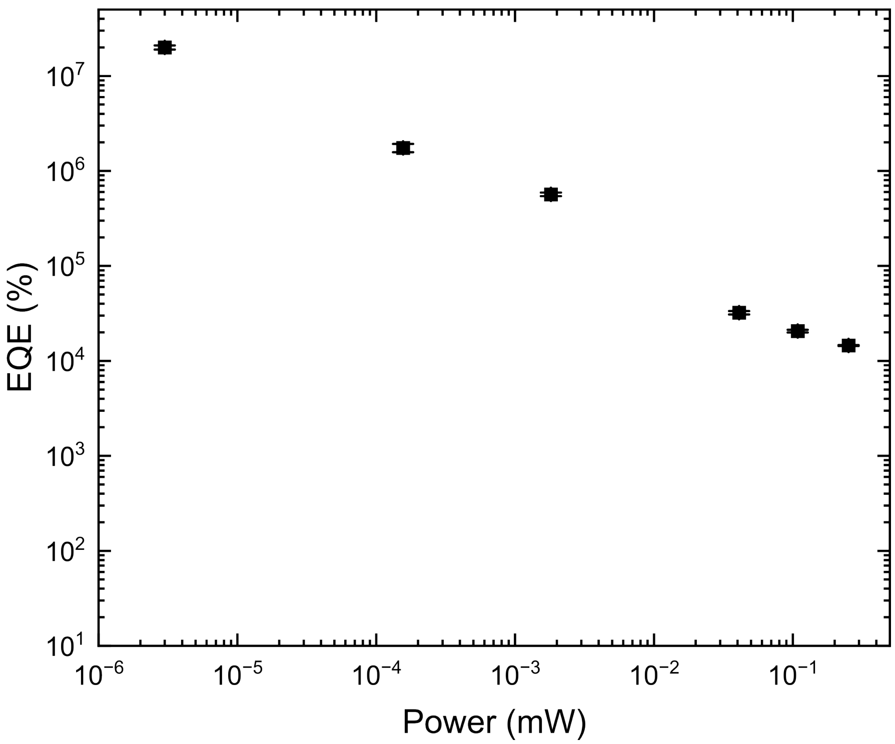

Figure S6: Calculated EQE of the PD under different optical powers. Data are presented as mean±SD (n=3).

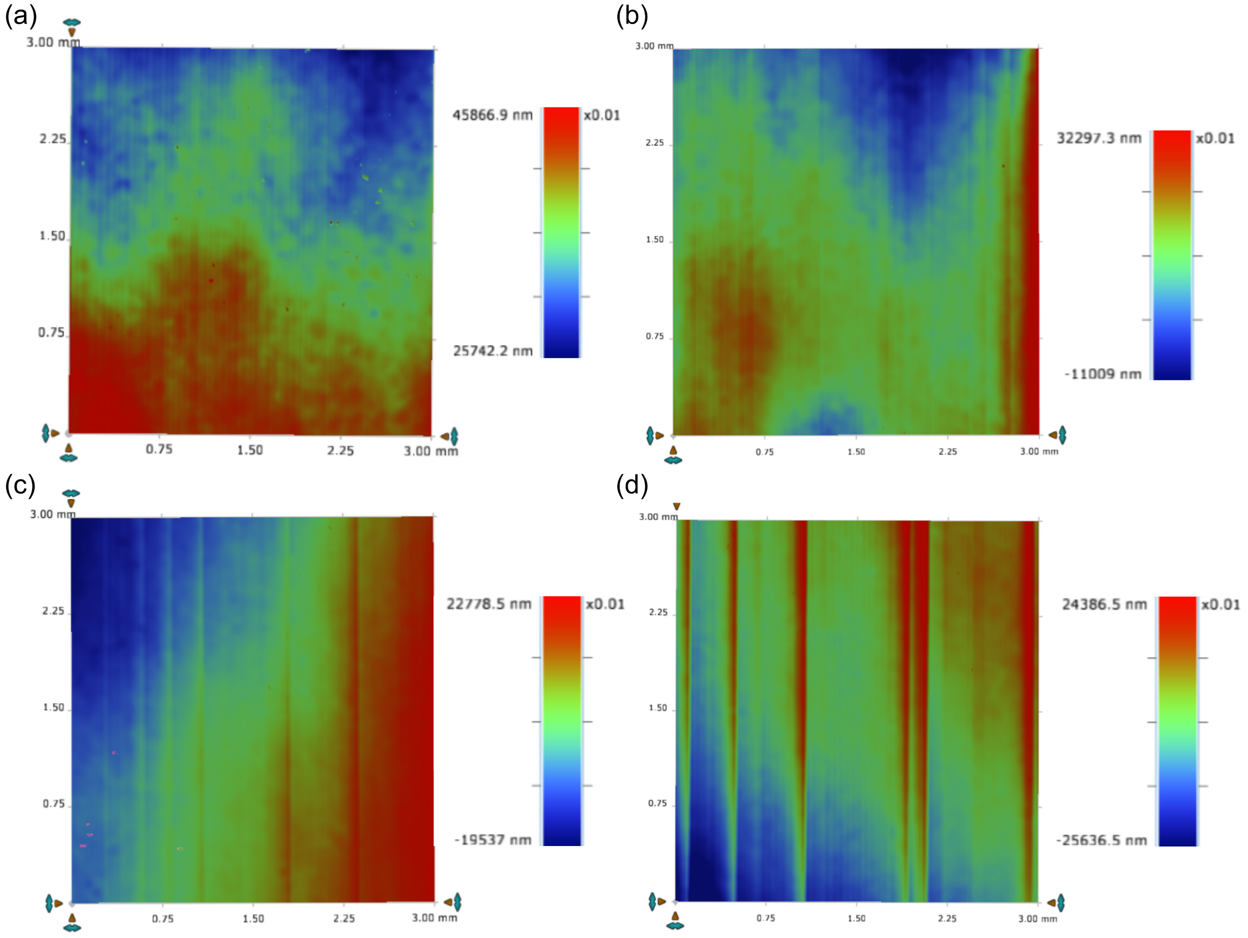

Figure S7: Three-dimensional mapping images of (a) paper substrate before bending, (b) paper substrate after bending at $L_0/L=1.18$, (c) paper substrate after 600 times bending at $L_0/L=1.15$, and (d) paper substrate after 1000 times bending at $L_0/L=1.15$.

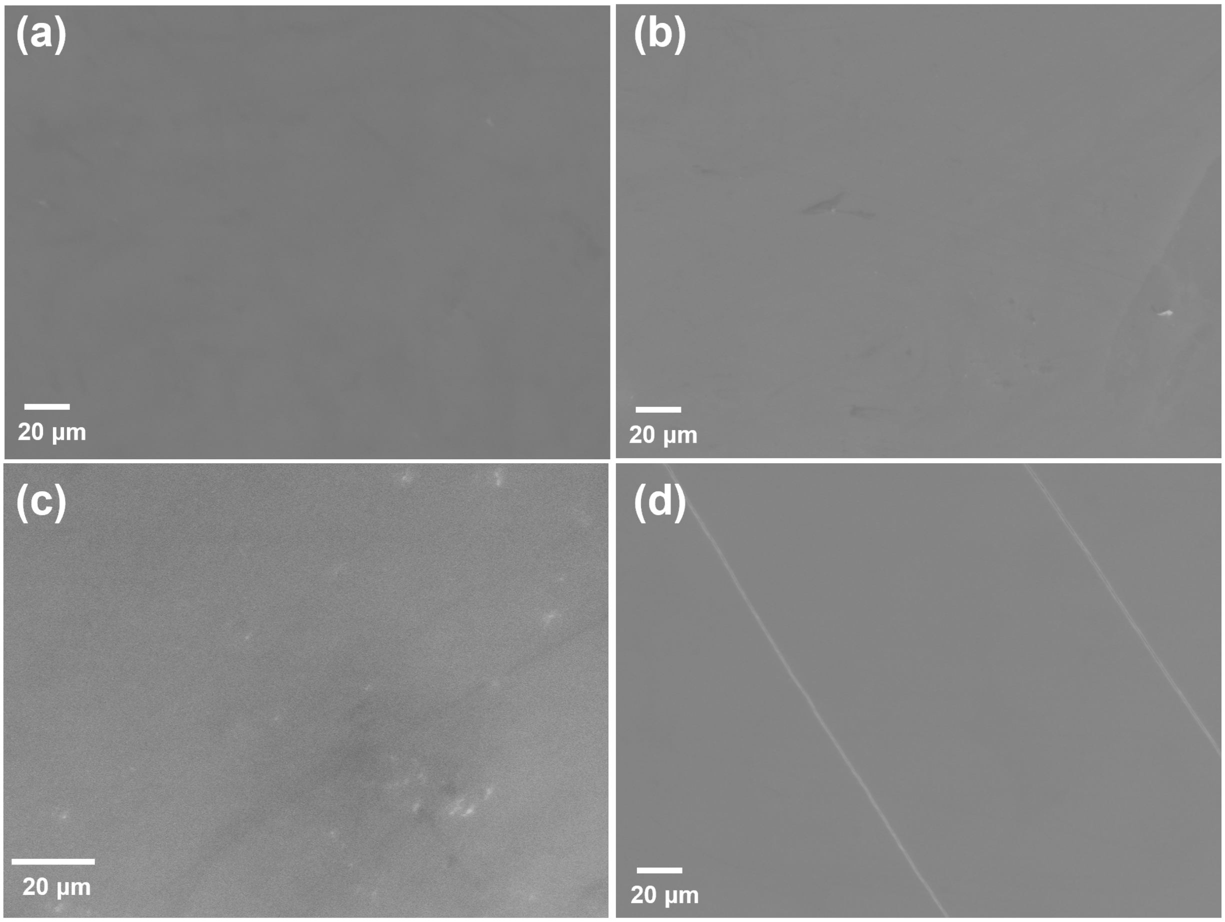

Figure S8: SEM images of (a) paper substrate before bending, (b) paper substrate after bending at $L_0/L=1.18$, (c) paper substrate after 600 times bending at $L_0/L=1.15$, and (d) paper substrate after 1000 times bending at $L_0/L=1.15$.

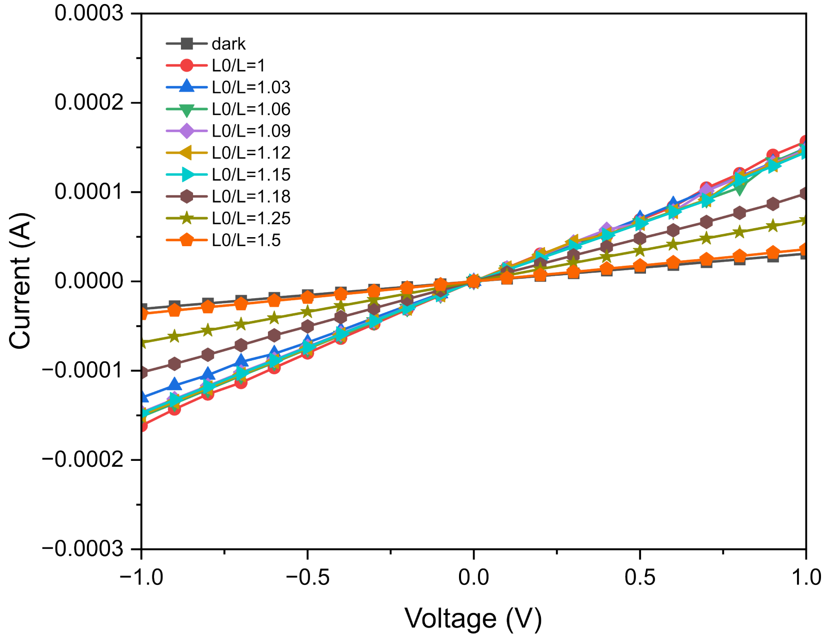

Figure S9: I-V curves of PD at different bending angles under the illumination of 0.2 mW input power at 520 nm incident laser at ambient conditions.

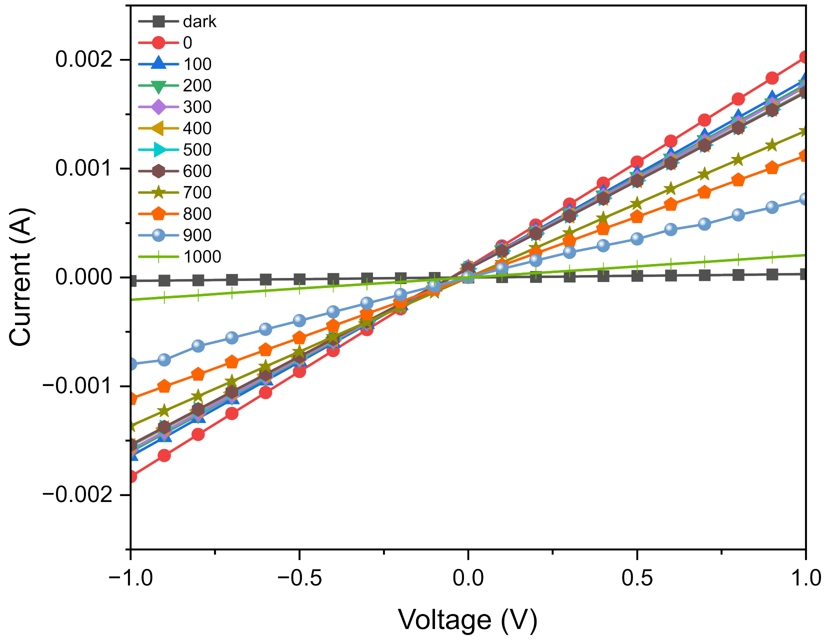

Figure S10: I-V curves of PD at 1.15 bending angle and different bending cycles under the illumination of 0.2 mW input power at 520 nm incident laser at ambient conditions.

To assess the reproducibility of our devices, we fabricated and tested six additional devices, all exposed to the same optical power (3 nW at 520 nm). The results (Fig. S11) demonstrated consistent performance across all devices, confirming the reliability and uniformity of the fabrication method using printed lithography.

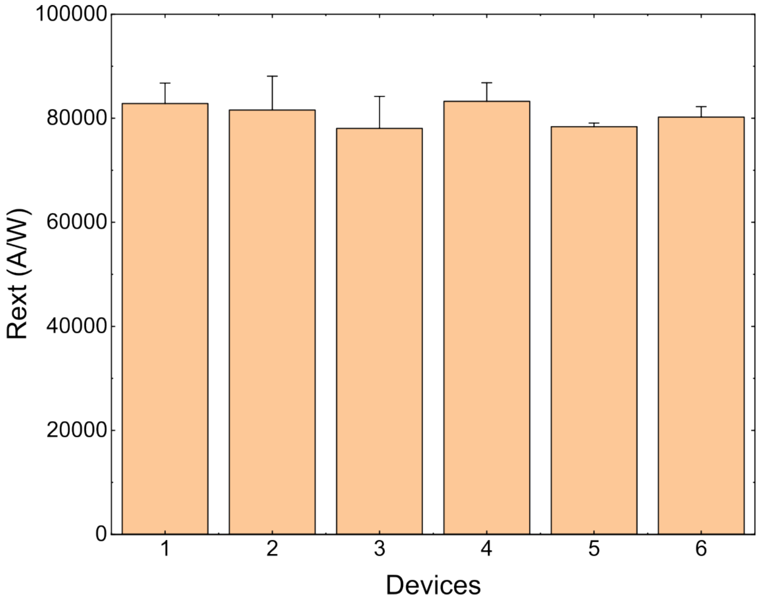

Figure S11: Repeatability and uniformity of PDs under 3 nW optical power. Data are presented as mean±SD (n=3).

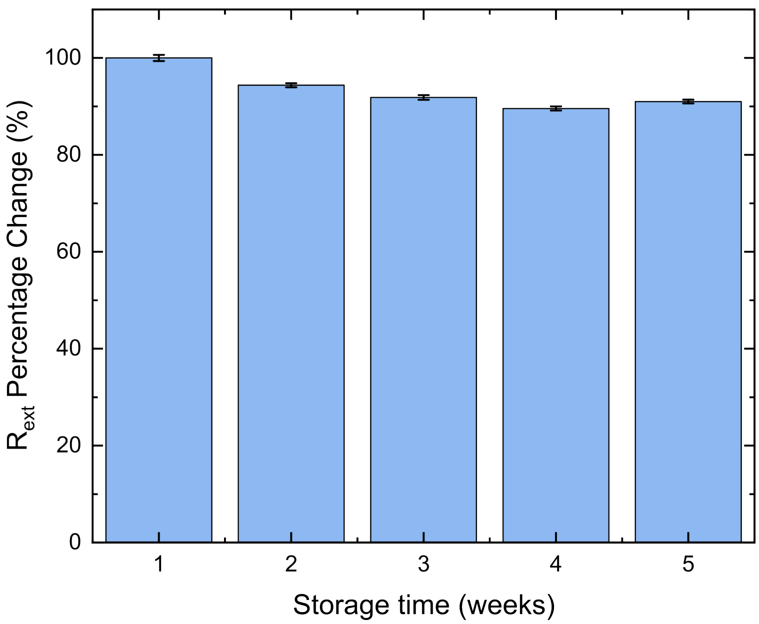

Figure S12: The long-term stability of $CsPbBr_3$ PQDs/graphene PD over five weeks. Data are presented as mean±SD (n=3).

References

(1) Li, S.-X.; Xu, X.-L.; Yang, Y.; Xu, Y.-S.; Xu, Y.; Xia, H. Highly deformable high-performance paper-based perovskite photodetector with improved stability. *ACS applied materials & interfaces* **2021**, *13*, 31919–31927.
